# mNGS facilitates the diagnosis of pediatric murine typhus: A case report

**DOI:** 10.1097/MD.0000000000047253

**Published:** 2026-01-16

**Authors:** Jia-Xin Duan, Hui Jian, Li Chang, Jie Teng, Shu-Yu Lai, Qiu-Xia Yang, Guang-Lu Che, Li-Li Luo, Fang Liu

**Affiliations:** a Department of Laboratory Medicine, West China Second University Hospital, Sichuan University, Chengdu, Sichuan, China; b Key Laboratory of Birth Defects and Related Diseases of Women and Children, Sichuan University, Ministry of Education, Chengdu, Sichuan, China; c Department of Pediatrics, West China Second University Hospital, Sichuan University, Chengdu, Sichuan, China.

**Keywords:** hemophagocytic lymphohistiocytosis, metagenomics next-generation sequencing, murine typhus, *Rickettsia typhi*

## Abstract

**Rationale:**

Murine typhus, caused by *Rickettsia typhi*, is a globally distributed flea-borne rickettsiosis. Although rarely recognized, it can trigger hemophagocytic lymphohistiocytosis (HLH), a life-threatening hyperinflammatory syndrome. Nonspecific febrile illness and atypical petechial eruptions frequently lead to delayed or missed diagnoses. This report aims to illustrate the diagnostic process and clinical implications of murine typhus-associated HLH in a pediatric patient, and to evaluate the utility of metagenomic next-generation sequencing (mNGS) as an unbiased detection tool for occult pathogens.

**Patient concerns:**

A 10-year-old child was admitted with unexplained recurrent fever and generalized petechiae, refractory to treatment at outside hospitals.

**Diagnoses:**

The patient was ultimately diagnosed with murine typhus-associated HLH caused by *R typhi*, based on a comprehensive diagnostic work-up.

**Interventions:**

Empirical dexamethasone was initiated promptly to control hyperinflammation. After mNGS confirmation, oral doxycycline was added for targeted anti-rickettsial therapy.

**Outcomes:**

The patient’s clinical status continued to improve, culminating in discharge.

**Lessons:**

Murine typhus-associated HLH should be considered in febrile children with unexplained cytopenias and petechiae. Early empiric HLH-directed immunosuppression followed by pathogen-specific therapy improves prognosis. mNGS provides a rapid, unbiased method to detect rare, overlooked pathogens and guide definitive treatment when conventional tests are negative.

## 1. Introduction

Murine typhus, caused by *Rickettsia typhi*, is a prevalent vector-borne disease transmitted by rodent fleas. The most common symptoms include fever, headache, rash, and arthralgia.^[1]^ If not promptly treated, it could escalate to severe complications such as meningoencephalitis, pneumonia, septic shock, and respiratory failure.^[2]^ Hemophagocytic lymphohistiocytosis (HLH) triggered by *R typhi* is a life-threatening syndrome.^[3]^ This can lead to respiratory insufficiency and multiorgan dysfunction.^[4]^ Clinical diagnosis of murine typhus-induced HLH can be challenging because HLH is associated with various infections, malignancies, and other triggers.^[5]^

A case of murine typhus-associated HLH presenting with sustained fever and generalized petechiae in a pediatric patient is described herein. Empiric dexamethasone promptly controlled HLH, and subsequent metagenomic next-generation sequencing (mNGS) uncovered *R typhi*. After being promptly treated with doxycycline, the patient made a swift recovery.

## 2. Case presentation

A 10-year-old patient was admitted to the hospital after experiencing repeated high fever for 7 days and generalized petechiae for 4 days. The patient complained of headache and dizziness, exhibited a diminished mental status, and experienced pain in both lower limbs at the onset of the illness. The patient’s guardians reported that petechiae emerged during the course of illness and subsequently spread diffusely across the body. After unsuccessful treatments at 2 hospitals, the patient was transferred to the hospital for further treatment on September 6, 2024.

The patient denied any prior infectious or other significant medical history. The family lives in a region where murine typhus is endemic. They kept poultry at home, and the patient had contact with the animals. They also claimed that they occasionally saw rats in their homes.

**Figure 1.**
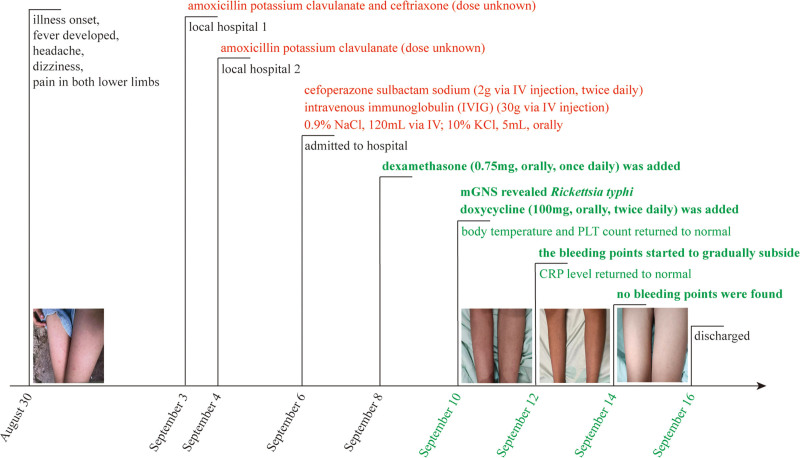
Timeline of the patient’s progress. The timeline illustrates key events in the patient’s illness with color-coded lines. Black indicates disease deterioration, red shows ineffective drug treatment, underlining marks turning points, and green signifies recovery.

Physical examination revealed moderately enlarged tonsils, hepatosplenomegaly, and petechiae in the cheeks and limbs. Routine examination revealed abnormal blood values, including elevated C-reactive protein (CRP), decreased white blood cell, hemoglobin, and platelet. Arterial blood gas analysis reported a hemoglobin concentration of 83 g/L. Detailed experimental data can be found in Table S1, Supplemental Digital Content, https://links.lww.com/MD/R168. Considering the patient’s medical background and laboratory findings, the primary diagnoses were established: sepsis, unidentified cause of the reduction in the 3 lineages of peripheral blood cells, electrolyte disturbances, and hypoalbuminemia. The patient received intravenous immunoglobulin (IVIG; 30 g via IV injection). Subsequently, fluid and electrolyte infusions were initiated (0.9% NaCl, 120 mL via IV; 10% KCl, 5 mL, orally). The subsequent dosing was adjusted based on laboratory results, and by September 10, the electrolyte imbalances had been corrected. Additionally, the patient was administered cefoperazone sulbactam sodium (2 g via IV injection, twice daily) for anti-infective purposes (Fig. 1).

**Figure 2.**
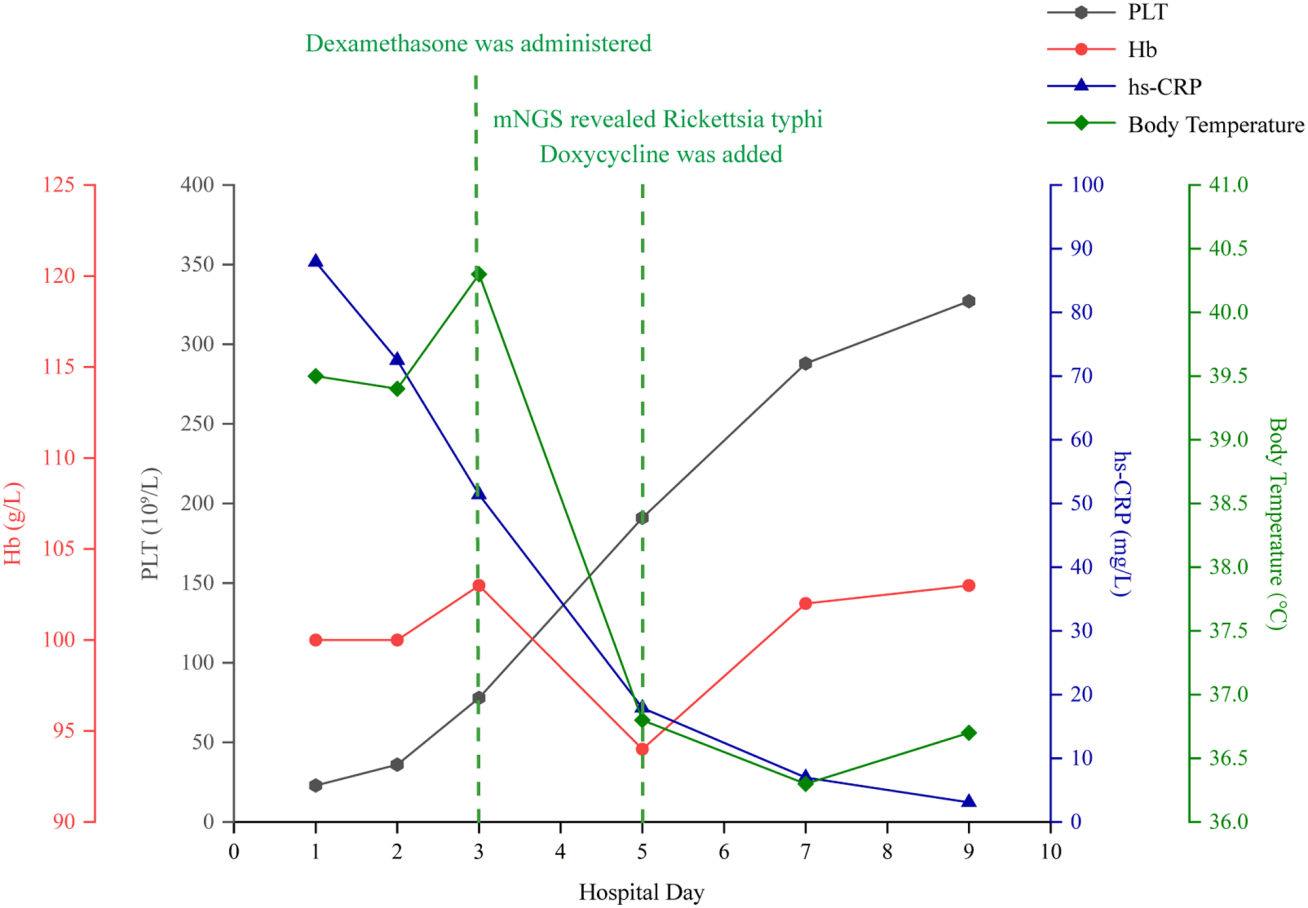
Clinical laboratory examination values during hospitalization. C-reactive protein (CRP, blue line) levels and body temperature (green line) rapidly decreased following the administration of dexamethasone. The blood platelet (PLT, gray line) count was obviously increased due to the treatment with dexamethasone. Following the administration of doxycycline, the patient’s PLT count, CRP levels, and body temperature progressively normalized. Hemoglobin (Hb, red line) remained low but steadily rose after doxycycline initiation and normalized during subsequent follow-ups. CRP = C-reactive protein, Hb = hemoglobin, PLT = platelet.

Serological examination yielded negative results for common pathogens, including hepatitis B, hepatitis C, human immunodeficiency virus, syphilis, Epstein–Barr virus, and Japanese encephalitis virus. Similarly, the blood, cerebrospinal fluid, and bone marrow cultures showed no growth. The timeline and images of this case are shown in Figure 1. The laboratory findings during her hospitalization are displayed in Figure 2.

**Figure 3.**
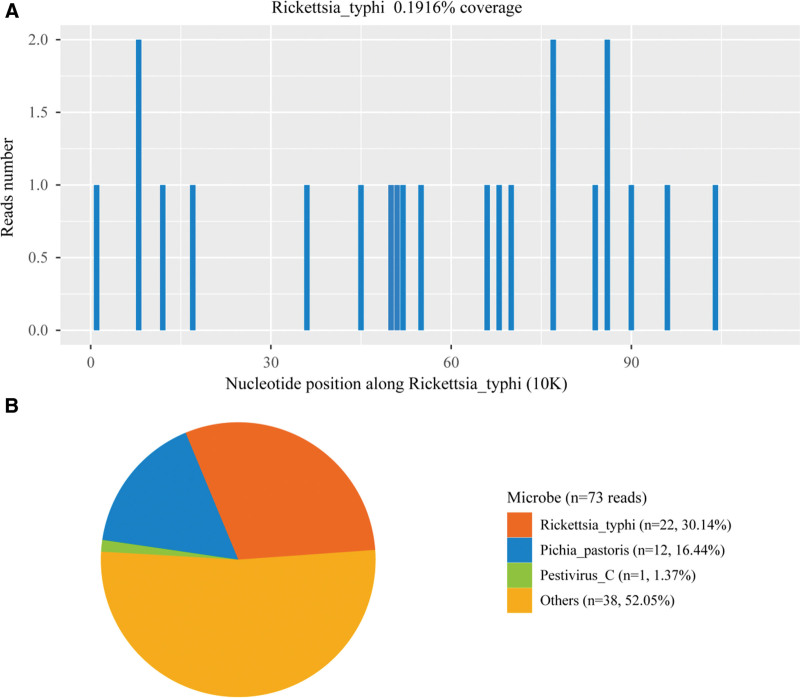
Comprehensive metagenomic next-generation sequencing (mNGS) profiling in plasma reveals *Rickettsia typhi (R typhi*) and diverse microorganisms. (A) Genomic mapping of *R typhi* sequence reads. (B) Raw read counts of all microorganisms identified by mNGS. After removal of human, unclassified and low-quality sequences, only *R typhi* (22 reads) fulfilled clinical criteria as the causative pathogen; all other signals are considered background or environmental contamination. mNGS = metagenomic next-generation sequencing, *R typhi* = *Rickettsia typhi*.

Fortunately, our attention quickly shifted to infection-associated HLH, given our previous experience with 2 cases of scrub typhus-associated HLH.^[6]^ On the third day of admission (September 8), the diagnosis was updated to HLH, which was again confirmed by the bone marrow smear results 4 days later. Dexamethasone (0.75 mg, orally, once daily) was initiated for the symptomatic anti-inflammatory treatment on the same day (Fig. 1). The patient experienced symptom relief, marked by a significant increase in platelet levels, an additional decline in CRP levels, and a progressive normalization of body temperature. On 8 September, an additional blood sample was collected for mNGS; 2 days later, 22 *R typhi*-specific reads were identified, corresponding to a genome coverage of 0.1916% and a relative abundance of 61.36% (Fig. 3). Plasma used for mNGS was subjected to reverse transcription real-time polymerase chain reaction validation. Detailed protocols are described in the Supplementary Materials, Supplemental Digital Content, https://links.lww.com/MD/R168. The diagnosis and treatment of murine typhus and infection-associated HLH are summarized in Table S2, Supplemental Digital Content, https://links.lww.com/MD/R168.

Consequently, the patient was diagnosed with murine typhus-associated HLH, and the antibiotic treatment was changed to cefoperazone sulbactam sodium (2 g via IV injection, twice daily) combined with doxycycline (100 mg, orally, twice daily; Fig. 1). As the patient’s condition improved, treatment targeting *R typhi* with doxycycline sped up the resolution of the petechial rash, resulting in noticeable improvement. The petechiae on the cheek skin gradually subsided, and there were a few scattered petechiae on both lower limbs (September 10), which decreased compared to before. After 4 days of treatment (September 14), no petechiae were found throughout the body. The symptoms gradually improved, and the blood and biochemical indicators gradually recovered. The patient was discharged on September 16, 2024. Comprehensive examinations were conducted during the follow-up visit, and all results indicated that the child’s condition remained normal. The patient is asymptomatic and perceives a complete return to premorbid baseline.

## 3. Discussion

A pediatric patient with recurrent fever and generalized petechiae who was admitted after unsuccessful treatment is reported. Pathogen infection-associated HLH was suspected after learning of mountain residence, poultry exposure, symptoms, laboratory results, and our clinical experience in treating such cases. The patient received an appropriate dose and duration of dexamethasone, which quickly alleviated HLH symptoms. Subsequently, the diagnosis of murine typhus-associated HLH was confirmed using mNGS, which detected 22 *R typhi* reads in the patient’s plasma sample. Doxycycline, an anti-*R typhi* drug, was added to the treatment regimen, which relieved the patient’s symptoms of generalized petechiae and eventually completely disappeared.

Murine typhus in pediatric patients typically presents with fever, anorexia, malaise, fatigue, and headache.^[7-9]^ Abdominal pain, diarrhea, and sore throat are also fairly common.^[1]^ Other symptoms, including vomiting, lymphadenopathy, conjunctivitis,^[10]^ localized myositis,^[9]^ and multisystem inflammatory syndrome,^[11]^ have also been reported. Maculopapular rashes, severe thrombocytopenia, and mild anemia have been reported in some cases.^[12,13]^ In addition to thrombocytopenia, laboratory abnormalities may include elevated CRP levels, hypoalbuminemia, elevated erythrocyte sedimentation rate, elevated transaminases, and increased neutrophil band count.^[7,8]^ Although the illness usually begins with mild symptoms, it can quickly progress to a severe state. In such cases, symptoms such as fever, headache, and poor mental response make it challenging for inexperienced clinicians to associate the condition with a specific pathogenic infection. There is no specific test for *R typhi* available at a local hospital with limited medical resources. Consequently, the diagnosis was overlooked, leading to a rapid progression to sepsis before the patient was admitted to the hospital.

In murine typhus, rash, which can manifest as macules, maculopapules, papules, and occasionally petechiae, is present in approximately half of the patients.^[2,14]^ At the onset of the illness, the patient presented with rashes on both lower limbs, which quickly disappeared. Upon admission, no typical rashes were observed; instead, there were non-blanching, non-protruding, non-itchy, and well-demarcated ecchymoses, previously described as petechiae in the medical history. Given that the patient’s family kept poultry at home and rats were occasionally seen, the early rashes were mistakenly attributed to common flea bites. After the patient returned home from the local hospital, a transition from rash to ecchymoses occurred without continuous medical observation. This lack of continuous monitoring contributed to the initial misinterpretation of the cause of the ecchymosis. Only after mNGS had confirmed *R typhi* as the causative agent could the entire disease course be retrospectively reconstructed. The ecchymoses that appeared later were due to the evolution of the initial rash.

As reported, in cases of HLH, when direct pathogen therapy is unavailable to control the disease, appropriate therapies such as dexamethasone could be life-saving.^[15]^ However, it is also crucial to promptly identify the infectious pathogen and administer doxycycline for targeted treatment to control the infection.^[16]^ In our study, the patient’s rapid recovery was primarily attributed to the timely administration of dexamethasone and swift adjustment of the treatment plan to incorporate doxycycline, following mNGS identification of the pathogen. In this case, the detection of a low rickettsial load was related to the timing of the blood sample collection. Prior to sample collection, the patient was treated with high-dose IVIG, dexamethasone (a potent corticosteroid), and the broad-spectrum antibiotic, cefoperazone-sulbactam. These interventions led to significant improvements in the patient’s condition. Subsequently, treatment targeting *R typhi* with doxycycline accelerated the resolution of petechial rash. This experience reaffirms the importance of considering HLH associated with specific pathogen infections in patients presenting with unexplained fever, significantly elevated CRP levels, and pancytopenia, particularly when there is a history of potential exposure. This also shows that clinicians’ comprehensive understanding of the various manifestations and related complications of unique pathogens is crucial for early diagnosis and for reducing mortality.

The diagnostic modalities for murine typhus include culture, nucleic acid amplification, and serology; however, each method has several limitations in practicality.^[17,18]^ Murine typhus is extremely rare in Chengdu. As a result, the hospital is unable to provide a Weil-Felix agglutination test, other immunofluorescence assays for *R typhi* infections, or PCR testing. As an unbiased pathogen detection technique, mNGS enables direct pathogen identification from clinical specimens, bypassing the requirement for predefined targets, and offers superior detection capabilities for non-cultivable pathogens compared to traditional cultivation methods.^[19,20]^ Given the high cost of mNGS and its potential financial burden on patients, this test was reserved for cases in which common viral infections such as Epstein–Barr virus had been excluded. Ultimately, the results of mNGS directly facilitate precise diagnosis and effective treatment, culminating in favorable patient outcomes.

## 4. Conclusion

Murine typhus-associated HLH should be entertained in any child with prolonged fever, cytopenias and atypical purpura even when a classic eschar is absent. After exclusion of primary immunodeficiency, concealed infectious triggers must be actively sought. In settings where rickettsioses are infrequent, routine laboratory tests are frequently insufficient; mNGS then serves as an unbiased, rapid screen for occult pathogens. Timely pathogen identification allows immediate targeted therapy, interrupts disease progression, reduces length of stay and secures an excellent outcome.

## Acknowledgments

The authors express their sincere gratitude to every member of the healthcare team whose dedication and expertise contributed to the care and management of these patients.

## Author contributions

**Conceptualization:** Li-Li Luo, Fang Liu.

**Data curation:** Jia-Xin Duan, Jie Teng, Shu-Yu Lai.

**Funding acquisition:** Li-Li Luo, Fang Liu.

**Investigation:** Jia-Xin Duan, Qiu-Xia Yang, Guang-Lu Che.

**Supervision:** Hui Jian, Li Chang, Li-Li Luo, Fang Liu.

**Writing – original draft:** Jia-Xin Duan.

**Writing – review & editing:** Hui Jian, Li Chang, Jie Teng, Shu-Yu Lai, Qiu-Xia Yang, Guang-Lu Che, Li-Li Luo, Fang Liu.

## Supplementary Material



## References

[1] Tsioutis C Zafeiri M Avramopoulos A Prousali E Miligkos M Karageorgos SA. Clinical and laboratory characteristics, epidemiology, and outcomes of murine typhus: a systematic review. Acta Trop. 2017;166:16–24. 27983969 10.1016/j.actatropica.2016.10.018

[2] Blanton LS. Murine typhus: a review of a reemerging flea-borne rickettsiosis with potential for neurologic manifestations and sequalae. Infect Disease Rep. 2023;15:700–16. 37987401 10.3390/idr15060063 PMC10660532

[3] Zamora Gonzalez RA Mayo MS Jeng AC. Severe flea-borne typhus complicated by hemophagocytic lymphohistiocytosis: a case report and review of literature. IDCases. 2024;36:e01955. 38646601 10.1016/j.idcr.2024.e01955 PMC11031783

[4] Leal-López VF Arias-León JJ Faccini-Martínez AA. Fatal murine typhus with hemophagocytic lymphohistiocytosis in a child. Rev Inst Med Trop São Paulo. 2020;62:e99. 33331518 10.1590/S1678-9946202062099 PMC7748055

[5] Poudyal BS Poudel B Tuladhar S Rijal SS Shrestha GS Joshi U. Hemophagocytic lymphohistiocytosis: an under-recognized and life-threatening condition. J Nepal Health Res Counc. 2023;20:794–6. 36974876 10.33314/jnhrc.v20i3.4315

[6] Jian H Yang Q-x Duan J-x. mNGS helped diagnose scrub typhus-associated HLH in children: a report of two cases. Front Public Health. 2024;12:1321123. 38784570 10.3389/fpubh.2024.1321123 PMC11111966

[7] Howard A Fergie J. Murine typhus in south texas children: an 18-year review. Pediatr Infect Dis J. 2018;37:1071–6. 29465481 10.1097/INF.0000000000001954

[8] Rees R Park C Long B Spencer S Sutter D. Murine typhus presenting with mucosal involvement. J. Pediatric Infect. Dis. Soc.. 2021;10:540–2. 33269795 10.1093/jpids/piaa138

[9] Mehta M Marek R Arthur C Starke J Dutta A. Localized myositis and transient encephalopathy as presenting symptoms in murine typhus. Pediatr Infect Dis J. 2024;43:e242–4. 10.1097/INF.0000000000004306 38451920

[10] Erickson T da Silva J Nolan MS Marquez L Munoz FM Murray KO. Newly recognized pediatric cases of typhus group rickettsiosis, Houston, Texas, USA. Emerg Infect Dis. 2017;23:2068–71. 29148369 10.3201/eid2312.170631 PMC5708246

[11] Dean A Asaithambi R Neubauer HC. Murine typhus in 5 children hospitalized for multisystem inflammatory syndrome in children. Hosp Pediatr. 2021;11:e61–5. 33431429 10.1542/hpeds.2020-005652

[12] Sarikloglou E Goula A Sidiropoulos C Tsolia M Papa A. Murine typhus with marked thrombocytopenia in a child in northern greece and literature review. Jpn J Infect Dis. 2018;71:368–9. 29848847 10.7883/yoken.JJID.2018.091

[13] Wulff J Margolin J Coleman NE Demmler-Harrison G Lam F Shah MD. A severe case of murine typhus presenting with anemia and severe thrombocytopenia. J Pediatr Hematol Oncol. 2018;40:e185–90. 29200167 10.1097/MPH.0000000000001024

[14] Caravedo Martinez MA Ramírez-Hernández A Blanton LS. Manifestations and management of flea-borne rickettsioses. Res Rep Trop Med. 2021;12:1–14. 33574726 10.2147/RRTM.S274724 PMC7873028

[15] Iaria C Colomba C Di Carlo P Scarlata F Tolomeo M Cascio A. Rickettsia typhi and haemophagocytic syndrome. Am J Trop Med Hyg. 2017;97:1632. 29140237 10.4269/ajtmh.17-0606 PMC5817786

[16] Henriques A Guerra M Ramalho AR Coimbra A Lima J. Peripheral axonal neuropathy in hemophagocytic lymphohistiocytosis secondary to rickettsia conorii infection. Cureus. 2024;16:e75598. 39803065 10.7759/cureus.75598 PMC11724739

[17] Stewart AG Stewart AGA. An update on the laboratory diagnosis of rickettsia spp. infection. Pathogens. 2021;10:1319. 34684267 10.3390/pathogens10101319 PMC8541673

[18] Trung NV Hoi LT Dien VM. Clinical manifestations and molecular diagnosis of scrub typhus and murine typhus, Vietnam, 2015–2017. Emerg Infect Dis. 2019;25:633–41. 30882318 10.3201/eid2504.180691 PMC6433017

[19] Chang L Che G Yang Q. Leishmania donovani visceral leishmaniasis diagnosed by metagenomics next-generation sequencing in an infant with acute lymphoblastic leukemia: a case report. Front Public Health. 2023;11:1197149. 37435524 10.3389/fpubh.2023.1197149 PMC10332309

[20] Lu Z Han J Wang Y. Diagnosing and reintegrating traceability of infectious diseases via metagenomic next-generation sequencing: study of a severe case of rickettsia japonica infection. Infect Med. 2024;3:100094. 10.1016/j.imj.2024.100094 PMC10912839 38444746

