# Supplementary material for: mNGS facilitates the diagnosis of pediatric murine typhus: A case report
Source: Medicine (Baltimore). 2026 Jan 16;105(3):e47253. doi: 10.1097/MD.0000000000047253 (PMC12826250; doi:10.1097/MD.0000000000047253)
Supplement: Supplementary file 1 [file medi-105-e47253-s001.docx]

**mNGS Protocol**

Blood for metagenomic next-generation sequencing (mNGS) was drawn into a Streck Cell-Free DNA BCT® tube. After centrifugation at 1600g for 5 minutes, 200 μL of plasma was extracted from 1 mL of whole blood, and DNA was subsequently extracted from the plasma. Subsequently, a DNA library was constructed and sequenced on the NextSeq 550 platform (Illumina, USA). Following removal of human host sequences, the remaining high-quality reads were aligned to the Microbial Genome Databases ([ftp://ftp.ncbi.nlm.nih.gov/genomes/](https://ftp.ncbi.nlm.nih.gov/genomes/)) with BWA.

These sequences were then compared against the NCBI genome database (https://blast.ncbi.nlm.nih.gov/Blast.cgi) and showed a well match with the Rickettsia typhi str. genome sequence (Accession: NC_006142.1; LS992663.1; CP003398.1; CP003397.1; AE017197.1). The alignment results demonstrated a Query Cover of 100%, an E-value of 4e-30, and a percent identity of 100%.

The data sets analyzed in this study are publicly available in the Genome Sequence Archive (GSA) repository, which can be accessed at https://ngdc.cncb.ac.cn/gsa. The specific accession number for the data is CRA020694. The data of the detailed sequence information is also available at http://ngdc.cncb.ac.cn, reference number PRJCA039981.

**RT-rtPCR Protocol**

Plasma used for mNGS was subjected to reverse transcription real-time polymerase chain reaction (RT-rtPCR) validation.

A standard RT-rtPCR was carried out as reported by William S Probert et al. (2024) ^1^ using the primers (Rtyp23S_F, 5′-GAAAGACCCCGTGAACCTTTACTA-3′ and Rtyp23S_R, 5′-CTAACGCCTCTGCTTCGCAG-3′) and the TaqMan probe (Rtyp23S_P, 5′-6-FAM-TGCACATTT-ZEN-GACTTCTAACACC-IABkFQ-3′).

DNA was extracted from 200 μL serum. PCR reaction mixture was prepared as follows: 1× One Step PrimeScript III RT-PCR master mix (Takara Bio USA, San Jose, CA), primers at 400 nM, probe at 300 nM. The nucleic acid input volume was 5 µL for a final reaction volume of 25 µL.

Reverse transcription, amplification, and fluorescence detection were carried out using the Applied Biosystems® 7500 Real-Time PCR System. The thermal cycling conditions were as follows: incubation at 53°C for 10 minutes, initial denaturation at 95°C for 2 minutes, followed by 45 cycles of denaturation at 95°C for 3 seconds, and annealing/elongation at 57°C for 40 seconds. Fluorescent readings were collected during the anneal/extension step at 57°C.

**Reference**

1. Probert WS, Quintana AC, Kjemtrup AM, Hacker JK. Duplex Reverse-Transcription Real-Time Polymerase Chain Reaction Assay Targeting 23S rRNA Single Nucleotide Polymorphisms for the Detection of Flea-Borne Rickettsioses. *The American Journal of Tropical Medicine and Hygiene*. 2024;111(3):569-574. doi:10.4269/ajtmh.23-0884

| Supplementary Table 1. Detailed laboratory test results of the patient from admission to discharge. | | | | | | | | | | | | | | | | | | | | | | |
| --- | --- | --- | --- | --- | --- | --- | --- | --- | --- | --- | --- | --- | --- | --- | --- | --- | --- | --- | --- | --- | --- | --- |
| Time | Hospital day | WBC (4.3-11.3*10^9/L) | Hb (110-146 g/L) | N (1.6-7.8*10^9/L) | PLT (100-450*10^9/L) | IL-2R (< 700 U/mL) | K (3.5-5.5 mmol/L) | Na (132-146 mmol/L) | P (1.45-2.1 mmol/L) | Ca (2.25-2.67 mmol/L) | Fe (7.8-32.2umol/L) | SF (10-291 ng/mL) | D-D (< 0.55 mg/L FEU) | FDP (< 5 ug/mL) | FIB (200-400 mg/dL) | TG (< 1.7 mmol/L) | ALB (38.0-54.0 g/L) | ALT (< 49 U/L) | AST (< 40 U/L) | LDH (120-246 U/L) | ALP (91.1-278.1 U/L) | ADA (4-24 U/L) |
| Sep.6 (admission) | 1 | 3.7 | 100 | 2.66 | 61 | / | 2.63 | 128.6 | 0.9 | 1.81 | 1.37 | / | 10.29 | 35.6 | 181 | 2.96 | 24 | 22 | 51 | 465 | 70 | 99.9 |
| Sep.7 | 2 | 5.6 | 100 | 4.59 | 36 | 2288.4 | 3.4 | 135.7 | 0.66 | 1.9 | 2.66 | 1486 | / | / | / | / | / | / | / | / | / | / |
| Sep.8 (application of dexamethasone) | 3 | 6.7 | 103 | 3.42 | 78 | / | / | / | / | / | / | / | / | / | / | / | / | / | / | / | / | / |
| Sep.10 (application of doxycycline) | 5 | 8.7 | 94 | 6.4 | 191 | / | 3.53 | 139.8 | 1.33 | 2.11 | 15.84 | / | / | / | / | 1.58 | 26 | 34 | 46 | 328 | 56 | 57.3 |
| Sep.12 | 7 | 7.3 | 102 | 4.42 | 288 | / | / | / | / | / | / | / | / | / | / | / | / | / | / | / | / | / |
| Sep.14 | 9 | 12.1 | 103 | 5.45 | 327 | / | 3.17 | 139.5 | 1.33 | 2.23 | 17.65 | 270.4 | / | / | / | 1.37 | 31.3 | 65 | 34 | 211 | 76 | 37.2 |
| Sep.15 | 10 | / | / | / | / | / | / | / | / | / | / | / | 0.54 | 1.2 | 101 | / | / | / | / | / | / | / |
| Sep.16 (discharge) | 11 | / | / | / | / | / | / | / | / | / | / | / | / | / | / | / | / | / | / | / | / | / |
| Sep. 21 | / | 16.2 | 111 | 8.93 | 300 | / | / | / | / | / | / | / | / | / | / | / | / | / | / | / | / | / |
| Oct. 6 | / | 18.9 | 127 | 11.66 | 251 | / | / | / | / | / | / | / | / | / | / | 0.72 | 41.4 | 45 | 27 | 206 | 97 | 15.4 |
| Oct. 20 | / | 12.8 | 135 | 4.99 | 156 | / | / | / | / | / | / | / | / | / | / | / | / | / | / | / | / | / |
| Nov. 15 | / | 8.5 | 134 | 4.29 | 215 | / | / | / | / | / | / | / | / | / | / | / | 41 | 35 | 28 | 252 | 116 | 20 |
| Notes: WBC: white blood cells; Hb: hemoglobin; N: neutrophil; PLT: platelet; IL-2R: interleukin-2 receptor; SF: serum ferritin; D-D: D-dimer; FDP: fibrin degradation products; FIB: fibrinogen; TG: triglycerides; ALB : albumen; ALT: alanine aminotransferase; AST: aspartate aminotransferase; LDH: lactate dehydrogenase; ALP: alkaline phosphatase; ADA: adenosine deaminase | | | | | | | | | | | | | | | | | | | | | | |

**Supplementary Table 2.** Diagnosis and treatments for murine typhus and infection-associated HLH

|  | Murine typhus # | Infection-associated HLH * |
| --- | --- | --- |
| Diagnosis | Clinical manifestations:  Patients typically present with fever and headache or rash. Other symptoms include: myalgia, anorexia, nausea, vomiting, abdominal pain, cough, and altered mental status;  Exposure history:  A history of flea exposure or residing in an area with a high rodent population;  Laboratory tests:   1. Weil-Felix test: OX_19_ titre ≥ 1:160 or a 4-fold increase; 2. Indirect fluorescent antibody (IFA) assay: IgM ≥ 1:40 or IgG ≥ 1:162 3. 0, or a 4-fold increase; 4. Positive PCR of *R.typhi* in clinical samples; 5. Isolation of *R.typhi* from clinical samples. | Presence of five of eight diagnostic criteria:  (1) Fever;  (2) Splenomegaly;  (3) Cytopenia in at least two lines (hemoglobin < 90g/L, neutrophil count < 1*10^9^/L, PLT count < 100*10^9^/L);  (4) Hyperferritinaemia ≥ 500ng/mL;  (5) Hypofibrinogenemia (≤ 150mg/dL) and/or hypertriglyceridaemia (≥ 3mmol/L);  (6) Haemophagocytosis in bone marrow, spleen, or lymph nodes;  (7) Low or absent NK cell activity;  (8) High soluble CD25 (≥ 2400U/ml). |
| Treatments | Doxycycline (first-line) ^1^  Azithromycin  Chloramphenicol | Dexamethasone  Cyclosporine  Etoposide |
| # A diagnosis of murine typhus can be confirmed if the clinical symptoms and exposure history are consistent, and if one of the laboratory diagnostic criteria is met. ([Clinical Overview of Murine Typhus \| Typhus Fevers \| CDC](https://www.cdc.gov/typhus/hcp/clinical-overview/clinical-overview-of-murine-typhus.html)); Doxycycline continues to be the preferred treatment for murine typhus in children, as recommended by the Pediatric Red Book ^2^.  * In infection-triggered HLH, high-dose IVIG therapy in combination with corticosteroids should be considered. Additionally, antimicrobial agents should be initiated immediately upon presentation ^3^. | | |

**Reference**

1. Newton PN, Keolouangkhot V, Lee SJ, et al. A Prospective, Open-label, Randomized Trial of Doxycycline Versus Azithromycin for the Treatment of Uncomplicated Murine Typhus. *Clinical Infectious Diseases*. 2019;68(5):738-747. doi:10.1093/cid/ciy563

2. Caravedo Martinez MA, Ramírez-Hernández A, Blanton LS. Manifestations and Management of Flea-Borne Rickettsioses. *Research and Reports in Tropical Medicine*. 2021;Volume 12:1-14. doi:10.2147/rrtm.S274724

3. Koumadoraki E, Madouros N, Sharif S, Saleem A, Jarvis S, Khan S. Hemophagocytic Lymphohistiocytosis and Infection: A Literature Review. *Cureus*. 2022;doi:10.7759/cureus.22411
